# Outpatients with psychotic disorders still need physical health-promotion

**DOI:** 10.1192/j.eurpsy.2022.1981

**Published:** 2022-09-01

**Authors:** D.M. Larsen-Kaasgaard, L. Stryhn, M.K. Sorensen, P. Munk-Jørgensen, P. Hjorth

**Affiliations:** 1 Institute of Regional Health Services Research, University of Southern Denmark, Unit For Psychiatric Research, Vejle, Denmark; 2 Psychiatric Research Academy, Mental Health Services Region Of Southern Denmark, Odense, Denmark; 3 University of Southern Denmark, Unit For Psychiatric Research, Department Of Clinical Research,, Odense C, Denmark; 4 Psykiatrien i region syddanmark, Lokalpsykiatrien Vejle, Vejle, Denmark; 5 Region of Southern Denmark, Department Of Psychiatry Vejle, Vejle, Denmark

**Keywords:** preventing, physical health, PSYCHOTIC DISORDERS

## Abstract

**Introduction:**

Premature death of people living with non-affective psychotic disorders are related to life-style somatic comorbidities. Current health-promoting treatment and care programs does not target people living with psychotic disorders and therefore prevention and treatment do not embrace the accompanying challenges.

**Objectives:**

To identify and explore outpatients with non-affective psychotic disorders who are not offered existing municipal health-promoting treatment and care programs despite having a need.

**Methods:**

Two hundred and six eligible outpatients from multiple sites of the Psychiatric Services in the Region of Southern Denmark were invited to participate. At last, 165 outpatients met the criteria and agreed to participate. A screening scheme was used to identify socio-economic characteristics, life-style related somatic comorbidities, medication status and consumption of cigarettes, drugs and alcohol. In outpatients’ medical records measured values and blood samples were obtained.

**Results:**

Almost four-fifths of the outpatients were in need of health promotion out of whom more than half were not offered a municipal health-promoting treatment and care program. One or more of the investigated somatic comorbidities was found in more than one-third of the outpatients. 15% had type-2-diabetes mellitus and 10% had cardiovascular disease. Two-fifths of the outpatients were smokers. Mean number of cigarettes per day was 19 (SD=10) for smokers. Mean BMI for men was 29 kg/m^2^ (SD=7) and 34 kg/m^2^ (SD=8) for women.

**Conclusions:**

In general, the outpatient’s state of health was poor. Many outpatients were not offered a municipal health-promoting treatment and care program despite having a need.

**Disclosure:**

No significant relationships.

